# Intravascular Polarimetry: Clinical Translation and Future Applications of Catheter-Based Polarization Sensitive Optical Frequency Domain Imaging

**DOI:** 10.3389/fcvm.2020.00146

**Published:** 2020-08-28

**Authors:** Kenichiro Otsuka, Martin Villiger, Seemantini K. Nadkarni, Brett E. Bouma

**Affiliations:** ^1^Wellman Center for Photomedicine, Harvard Medical School, Massachusetts General Hospital, Boston, MA, United States; ^2^Institute for Medical Engineering and Science, Massachusetts Institute of Technology, Cambridge, MA, United States; ^3^Department of Cardiology, Erasmus University Medical Center, Rotterdam, Netherlands

**Keywords:** optical coherence tomography, polarimetry, atherosclerosis, collagen, smooth muscle cells, macrophage, neoatherosclerosis, drug-eluting stent

## Abstract

Optical coherence tomography (OCT) and optical frequency domain imaging (OFDI) visualize the coronary artery wall and plaque morphology in great detail. The advent of these high-resolution intracoronary imaging modalities has propelled our understanding of coronary atherosclerosis and provided enhanced guidance for percutaneous coronary intervention. Yet, the lack of contrast between distinct tissue types and plaque compositions impedes further elucidation of the complex mechanisms that contribute to acute coronary syndrome (ACS) and hinders the prospective identification of plaques susceptible to rupture. Intravascular polarimetry with polarization-sensitive OFDI measures polarization properties of the coronary arterial wall using conventional intravascular imaging catheters. The quantitative polarization metrics display notable image contrast between several relevant coronary plaque microstructures that are difficult to identify with conventional OCT and OFDI. Tissues rich in collagen and smooth muscle cells exhibit birefringence, while lipid and macrophages cause depolarization. In this review, we describe the basic principles of intravascular polarimetry, discuss the interpretation of the polarization signatures, and outline promising avenues for future research and clinical implications.

## Introduction

Coronary artery disease is a chronic inflammatory disease that in its most fatal complication provokes acute coronary syndrome (ACS) and in the long term leads to heart failure, causing immense disease burden, and economic cost worldwide (1–3). Substantial research efforts have been devoted to prospectively identifying “vulnerable plaques” that are prone to rupture and causing ACS with the goal to improve clinical therapy (4, 5). The ability to identify thin-capped fibroatheromas (TCFAs), heralded as the archetypical vulnerable plaque, has been a driving motivation for the development of intracoronary optical coherence tomography (OCT), and optical frequency domain imaging (OFDI)^1^ (6). These intravascular imaging methods visualize the subsurface microstructure of the arterial wall and atherosclerotic lesions with high spatial resolution (10 μm axial; 20–40 μm lateral), using light in the near infrared wavelength range (6). Today, OCT facilitates guiding percutaneous coronary intervention (PCI) with better physiological outcomes than using coronary angiography alone (7, 8), and has been shown to assess functional stenosis severity more accurately than intravascular ultrasound (IVUS) (9).

However, the majority of TCFAs identified by OCT (10–12) or virtual-histology IVUS (13–15) do not cause any acute events, calling into question the established structural criteria of the “vulnerable plaque” (5, 16, 17). Plaque composition has been identified as an additional critical factor of a lesion's susceptibility to precipitate ACS (4, 14, 18–20). Also, it is now a well-accepted concept that plaque rupture and erosion frequently occur silently without causing clinical symptoms, emphasizing the crucial role of vascular healing in determining the fate of a lesion (1, 21). Therefore, the mechanisms that impair vascular healing are increasingly investigated to explain disease progression and the development of ACS (22–24). Following stent implantation, vascular healing and tissue response play a similar decisive role in defining the risk of stent failure and future complications (25–28). Thus, there is an urgent need for imaging methods that afford refined insight into a lesion's make-up, composition, and healing, in order to address pertinent questions regarding the pathophysiology of atherosclerosis and to ultimately improve the risk stratification and management of patients with coronary artery disease.

Near-infrared spectroscopy, near-infrared fluorescence, and photoacoustic imaging assess aspects of coronary plaque composition, but depend on integration with intracoronary OCT or IVUS for structural imaging (29, 30). This multimodality approach requires significant modifications of the imaging catheter. To benefit from existing hardware and simplify clinical translation, we have been extending the capabilities of intravascular OCT by advancing polarization sensitive (PS-) OCT (31–33). Intravascular polarimetry (IVP) with PS-OCT measures the depth-dependent polarization state of the light scattered by tissue and provides spatially resolved maps of tissue birefringence and depolarization (34–36). IVP employs existing imaging catheters and can be performed with only minor modifications of current clinical intracoronary OCT systems (34, 36). The microscopic structure and organization of the arterial wall influence the polarization of the infrared light used by OCT, providing a compelling contrast mechanism. Birefringence is elevated in tissue with fibrillar architecture, such as interstitial collagen or smooth muscle cells (SMC), which play a critical role in plaque stability and vascular healing. Microscopic PS-OCT has been used early on to leverage this contrast mechanism (37–39), and birefringence measured in human aortic plaques *ex vivo* positively correlated with thick collagen fibers and SMCs (39). Despite its demonstrated potential, polarimetry with catheter-based PS-OCT proved challenging because of the dynamic variation of the polarization states transmitted through the rotating catheter (40) and polarization distortions induced by system components (41). We developed a reconstruction method that mitigates the resulting artifacts (42) enabling, for the first time, measurement of depth-resolved birefringence through intravascular imaging catheters, first *ex vivo* (34) and then in patients (35, 43–45). Measurement of the polarization states of the light returning from the tissue also permits evaluation of depolarization (46–48). Depolarization complements birefringence and provides the polarimetric characterization of tissues rich in lipid, cholesterol crystals, and macrophages (34–36).

IVP provides quantitative metrics of tissue polarization properties measured through conventional intravascular imaging catheters. Birefringence and depolarization complement the conventional tomograms of tissue microstructure with additional insights into tissue organization and composition that are highly relevant for studying the progression of coronary atherosclerosis and stratifying the risk of individual lesions. Here, we review the basic principles, clinical translation, and future prospects of IVP, and discuss how it may help advance our understanding of coronary artery disease.

## Working Principle of IVP With PS-OCT

**Figure 1:**
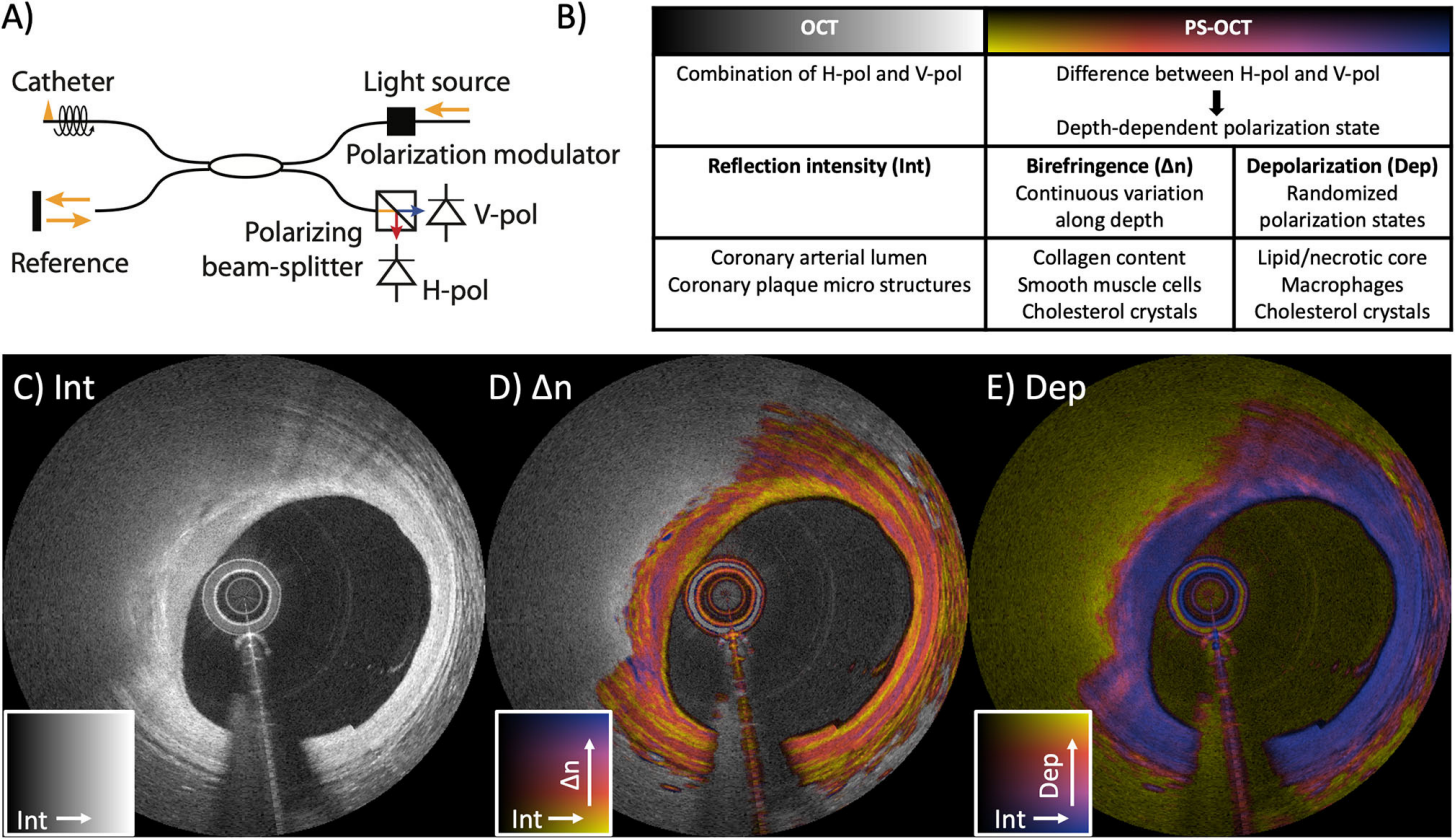
Working principle of intravascular polarimetry (IVP) with polarization-sensitive optical coherence tomography (PS-OCT). IVP is compatible with current intravascular imaging catheters and enables measurements of tissue polarization properties, together with the conventional reflection intensity. **(A)** The only addition to conventional OCT for enabling PS measurements is a polarization modulator that alternates the polarization state of the light incident on the tissue between depth scans. **(B)** Analyzing the spatial variation of the detected states allows reconstruction of birefringence (Δn) and depolarization (Dep), in addition to the conventional reflection intensity image (Int). **(C–E)** IVP of a lipid-rich plaque imaged in the left anterior descending coronary artery of a 75-year-old male patient. **(C)** Intensity (Int) of the reflection signal visualizing subsurface plaque morphology in a conventional logarithmic gray scale. **(D)** Birefringence (Δn) in color hue, overlaid on the reflection signal in brightness, revealing a variety of birefringence features. Birefringence is displayed only in areas of low depolarization. **(E)** Depolarization (Dep) in color hue, overlaid on the reflection signal, showing areas of pronounced depolarization. Reproduced from Villiger et al. (34).

Light is a transverse electromagnetic wave that oscillates in a plane orthogonal to the propagation direction of the beam. The oscillation pattern of the fields within this orthogonal plane defines the polarization state of the light, which may be altered by the optical components of the imaging system and catheter or by the vessel wall. Because the fiber-optic probe rotates inside the imaging catheter, the polarization state of the transmitted light varies dynamically. OCT measures the interference between back-reflected sample light and a reference beam, which has its own defined polarization state. Only light with identical polarization states can interfere. To ensure that the sample signal is detected independently of its specific polarization state, conventional OCT systems use two detection channels with orthogonal polarization states (Figure 1). Both the sample and the reference light are split between the two channels. The reference signal is adjusted to provide equal power in both channels and the sample light is split according to its specific polarization state. Conventional OCT tomograms combine the two polarization channels to reveal the reflection intensity as a function of depth independent of polarization effects and catheter rotation.

PS-OCT analyzes the ratio and phase difference between the signals in the two detection channels to recover the polarization state of the detected sample light as a function of the depth it traveled into the tissue (33). To obtain a complete characterization of the polarization effects of the sample independent of the transmission through the optical fiber, fiber-based PS-OCT furthermore modulates the polarization state of the illumination between consecutive depth-scans (49), although recent work suggests that a single input state may suffice (50). Tissue with fibrillar architecture, such as interstitial collagen or layered arrays of arterial SMCs, exhibits birefringence (Δn), an optical property that results in a differential delay, or retardance, between light polarized parallel to the tissue fibrillar components and light having a perpendicular polarization. The general polarization state of the illumination is a superposition of the parallel and perpendicular components and results in a continuous change of the light's polarization state as it propagates through the birefringent tissue. The experienced retardance is strongest for fibrillar components aligned in a plane orthogonal to the probing beam's direction and vanishes for fibrous tissue that align with the beam's axis. By analyzing the rate of change of the polarization states along depth, we compute a quantitative measurement of the effective depth-resolved tissue birefringence Δn. This is achieved by using our robust reconstruction strategy (42) that mitigates artifacts (41) originating from the polarization effects of the imperfect system components.

The majority of developments in PS-OCT have been focused on employing birefringence as an additional tissue contrast mechanism. Yet, PS-OCT can also measure depolarization to complement birefringence for the polarimetric characterization of tissue (46, 47, 51). In contrast to birefringent tissue, which induces a continuous change of the polarization states along depth but maintains high correlation along the lateral directions, depolarization corresponds to a random change of the polarization states in adjacent resolution volumes along all spatial directions. Depolarization is caused by multiple scattering of light, or by randomly oriented structures with polarization-dependent scattering, as caused by lipid droplets that exceed the size of the wavelength used for OCT or small cholesterol crystals, respectively. Hence, in atherosclerotic tissues, depolarization is observed in plaque regions that are rich in lipid, cholesterol crystals, or macrophages. Depolarization evaluates this randomness in a small neighborhood around each pixel as the ratio of the depolarized signal intensity to the total signal intensity, corresponding to 1 minus the degree of polarization (34, 48). Depolarization ranges from 0 for completely polarized light without any randomness to 1 for completely random polarization states. In addition to tissue-induced depolarization, regions where the OCT signal falls to the noise floor also appear depolarizing, requiring special consideration when interpreting the depolarization signal.

Displaying both the conventional and polarization channels available to IVP can be conveniently achieved using a color map that encodes the polarization metric as color hue and the conventional reflection signal as brightness. In all figures throughout this review, color maps display the range of 0-1.8 × 10^−3^ for birefringence, and 0-0.5 for depolarization (Figure 1). Given that the randomization of the polarization states frustrates a reliable measure of tissue birefringence, we display the conventional grayscale reflection tomogram in areas of increased depolarization in the birefringence maps.

## Characterization of Plaque Microstructures With IVP

Birefringence and depolarization are polarization metrics that enable quantitative analysis of intrinsic optical tissue properties related to the composition of coronary plaques. In order to identify the respective tissue components that cause observable polarization effects, we compared polarization properties measured with IVP in human cadaveric coronary arteries with matching histology (34). Birefringence maps reinforce several features that are already discernible in the OCT intensity image. As shown in Figure 1, the tunica media appears as a pinkish band with high birefringence, frequently bordered by fine yellow lines of low birefringence at the locations of the internal and external elastic laminae. Conventional OCT images, in comparison, show less pronounced contrast between the intima and the tunica media. Furthermore, fibrous tissue often exhibits heterogeneous birefringence maps across layers that appear homogeneous in the OCT intensity images. In Figure 1D, the fibrous cap features heterogeneous birefringence in the region of 9–12 o'clock. The depolarization map reveals low depolarization throughout the full thickness of the vessel wall in areas of adaptive intimal thickening, and abrupt strong depolarization in the lipid-rich area below the fibrous cap.

**Figure 2:**
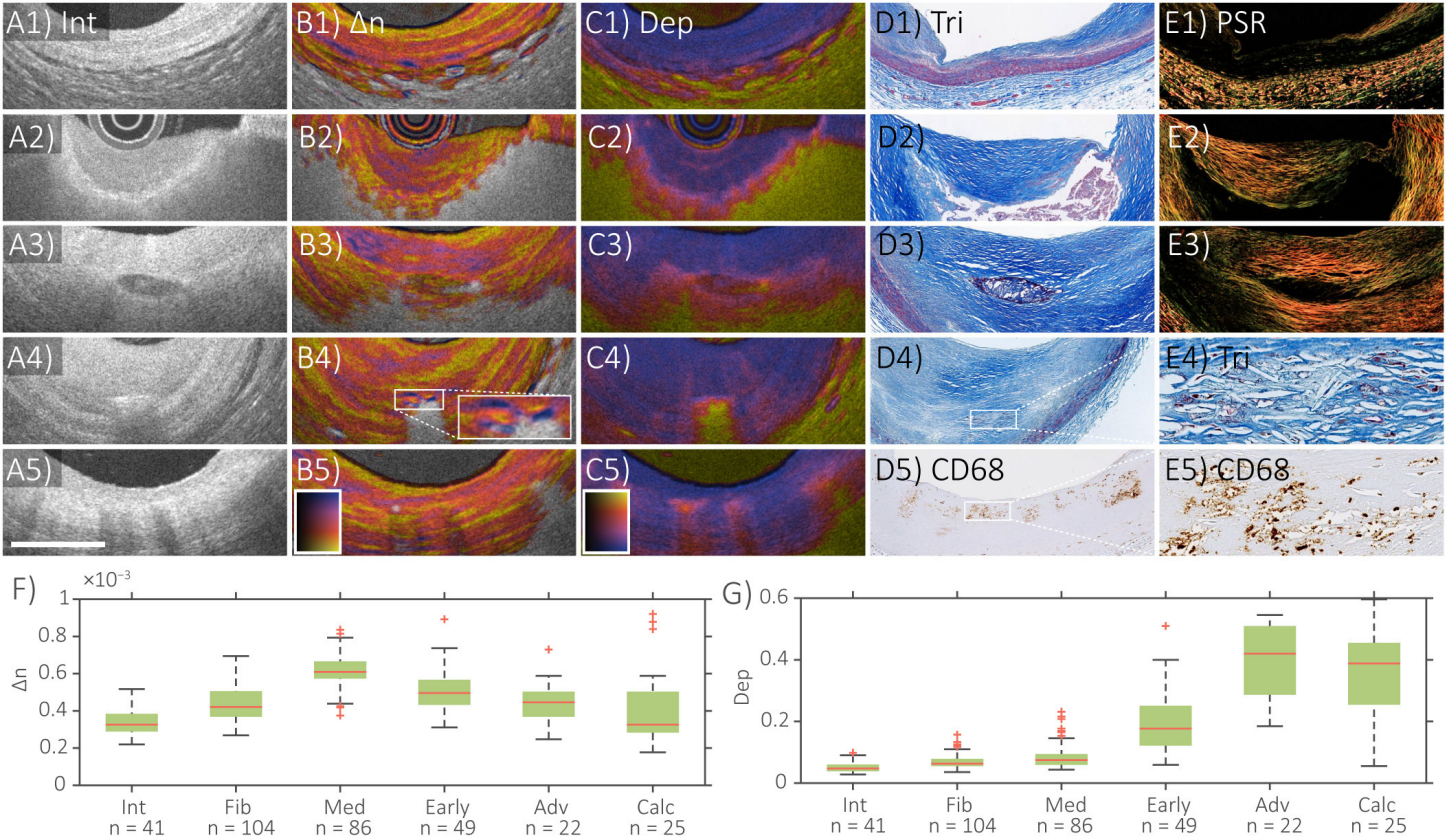
Distinct polarization features of atherosclerotic human coronary arteries *ex vivo*. IVP of **(A1–E1)** normal artery, **(A2–E2)** fibroatheroma, **(A3–E3)** fibrocalcific plaque, **(A4–E4)** cholesterol crystals, and **(A5–E5)** macrophages, with matching histology. **(F)** Median and quartile birefringence (Δn) values in regions with depolarization <0.2 across tissue types. **(G)** Median and quartile depolarization (Dep) values across tissue types. Scale bar, 1 mm. Int, intensity; Δn, birefringence; Dep, depolarization; Tri, trichrome staining; PSR, Picrosirius red staining; In (F), Int, intima; Fib, fibrous tissue; Med, tunica media; Early, early lesions with dispersed lipid; Adv, advanced lesions with lipid pools or necrotic core; Calc, calcifications. Adapted from Villiger et al. (34) and Otsuka et al. (36).

Figure 2 displays polarization features of several coronary plaque components along with matching histology, as well as measurements of polarization properties of individual tissue types [from (34)]. Quantitative analysis of segmented tissue types revealed the highest birefringence in the tunica media, followed by intimal regions containing fibrous tissue (Figure 2F). Consistent with a previous microscopic PS-OCT study (39), these observations suggest that collagen content and SMCs are the main source of the birefringence measured with IVP.

The highest depolarization was observed in segmented tissue corresponding to advanced lesions with a lipid/necrotic core (Figure 2G). Whereas conventional OCT lacks the ability to identify the presence of a necrotic core, and hence to differentiate between fibroatheroma and pathological intimal thickening (52, 53), depolarization offers additional insight that may enable diagnosis of fibroatheromas containing necrotic cores. In addition to the tissues and plaque components already discussed, Figure 2 also shows examples of the IVP signatures of a calcification, cholesterol crystals (CCs), and macrophage accumulations. The OCT intensity image depicts calcifications as signal poor or heterogeneous regions with sharply delineated borders (54), while their birefringence is generally lower and the depolarization slightly higher than in fibrous tissue (A3–E3 in Figure 2). However, their polarization properties also depend on the presence of lipid in the surrounding tissue. Clinical studies have demonstrated that CCs identified by conventional OCT are related to coronary plaque vulnerability in patients (55–58). CCs appear as thin, linear regions of high intensity signal, usually found in the fibrous cap or even within the necrotic core (54). Confusion of CCs with microcalcifications, however, is possible, since they can cause similar reflection signals (59). Consistent with the known birefringence property and dimensions of CCs, plaque regions containing small disordered CCs depolarize and areas with larger or aligned CCs additionally cause a birefringence signature (34) (A4–E4 in Figure 2). IVP may, therefore, improve the objective identification of CCs. In conventional OCT images, macrophage infiltration in the fibrous cap causes confluent punctate regions or signal-rich, distinct, and bright spots that exceed the intensity of background speckle noise (54, 59). With IVP, subtle depolarization was additionally observed in superficial regions infiltrated by macrophages (A5–E5 in Figure 2), which may help to automatically detect and quantify the presence of macrophages.

## *In vivo* Repeatability of IVP

We conducted the first-in-man pilot study of IVP at the Erasmus University Medical Center, the Netherlands, to investigate the robustness of IVP in a clinical setting. The quantitative metrics for tissue characterization provided by IVP are only meaningful if they can be evaluated repeatedly and robustly in the coronary arterial wall in patients. IVP employs commercialized OFDI catheters (FastView™, Terumo Corp., Tokyo, Japan) in combination with a custom console and the imaging procedure is identical to that using a conventional OFDI system (35, 43). To evaluate the reproducibility of the conventional backscatter intensity, the birefringence, and the depolarization signals, we compared each spatial location of 274 matching cross-sectional images among repeated pullbacks imaged in the coronary arteries of 30 patients (44). Bland-Altman analysis demonstrated best agreement for birefringence, followed by backscatter intensity, and depolarization. Pearson's correlation analysis confirmed highest correlation for birefringence (*r* = 0.86), preceding backscatter intensity (*r* = 0.83), and depolarization (*r* = 0.78). This finding confirmed that IVP provides reliable and repeatable measurement of tissue birefringence and depolarization through rotating catheters in a clinical setting and can serve for studying the polarization properties of coronary atherosclerosis in patients.

## IVP of Coronary Plaques in Patients

**Figure 3:**
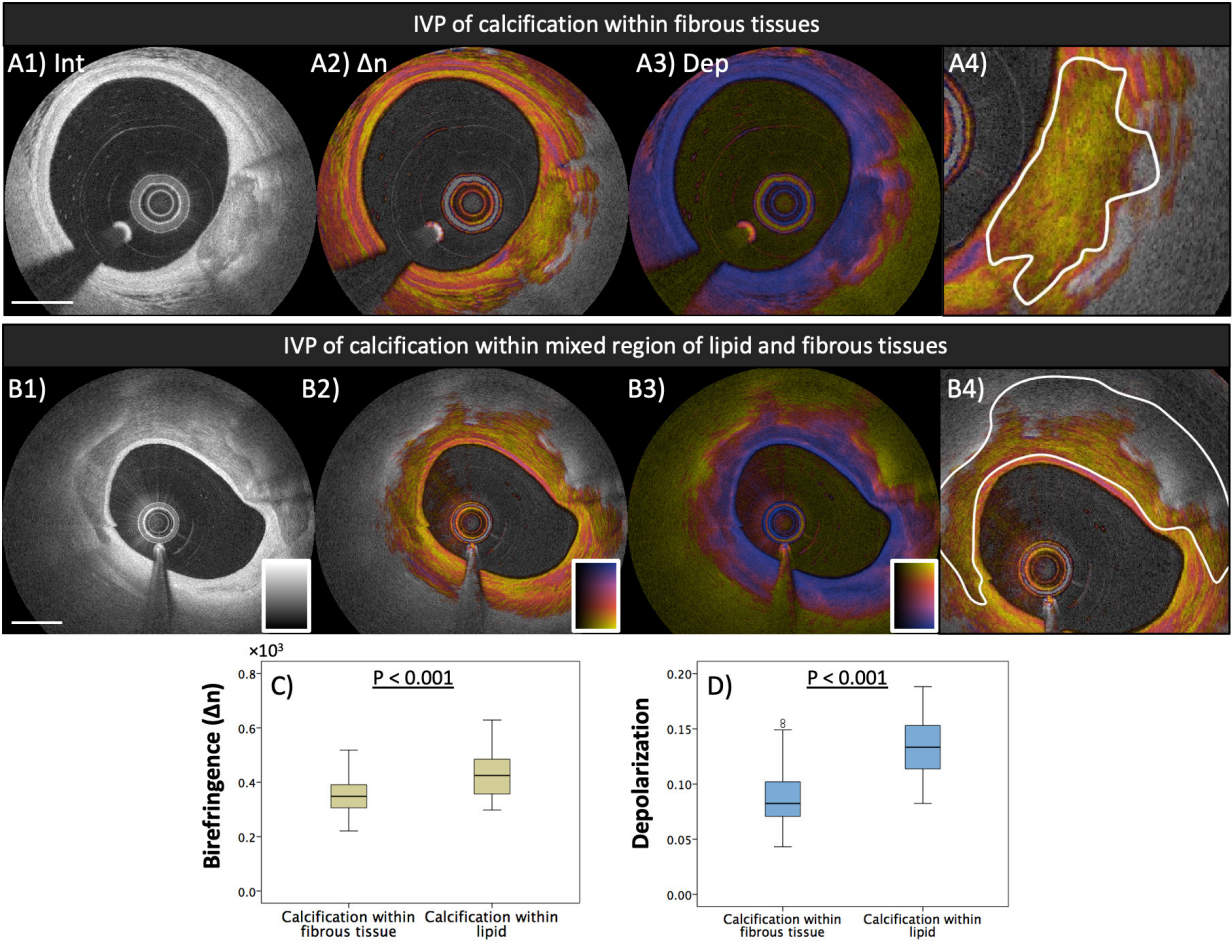
Polarization properties of calcification. Median birefringence and depolarization were measured in calcified areas, manually segmented in the intensity images. Segmented areas were classified according to the presence or absence of lipid in the surrounding lesion (calcification within fibrous tissue or calcification within lipid). **(A1–A4)** IVP of calcification in fibrous tissue and segmentation of the calcified area. **(B1–B4)** Calcification in lipid-rich tissue. **(C)** Calcified areas in fibrous tissue exhibit lower birefringence than those in lipid-rich lesions (*p* < 0.001). **(D)** Higher depolarization was observed in calcified areas in lipid-rich tissue than in those located in fibrous tissue (*p* < 0.001). Adapted from Otsuka et al. (35).

Motivated by the robust results of IVP measurements in a clinical setting, we investigated the quantitative polarization features of different plaque types, classified using the conventional OCT morphological appearance, among the plaques found in 30 patients with ACS (*n* = 12) and stable angina pectoris (SAP, *n* = 18) (35). Coronary plaques with a greater lipid content featured reduced birefringence and pronounced depolarization. We also further investigated the polarization features of coronary calcification measured with IVP. Calcification of the coronary artery has been shown to serve as a robust surrogate marker of coronary risk and is related to disease burden of coronary atherosclerosis (60–63). The presence of spotty calcification in coronary atheromatous plaques has been shown to be associated with the high-risk plaque morphology responsible for ACS (60, 62), while dense calcification appeared as a marker of lesion stability (61). Figure 3 shows IVP of two examples of intimal calcification together with the results from investigating all encountered calcifications within the analyzed cross-sections imaged in the 30 patients. In this sub-analysis, we observed that calcifications in fibrous regions featured lower birefringence compared to those in lipid-rich regions (*p* < 0.001). Although the possible clinical implication of the polarization features within calcifications measured with IVP remains unknown, we anticipate that IVP will provide further insight into the association of calcification and plaque stability.

**Figure 4:**
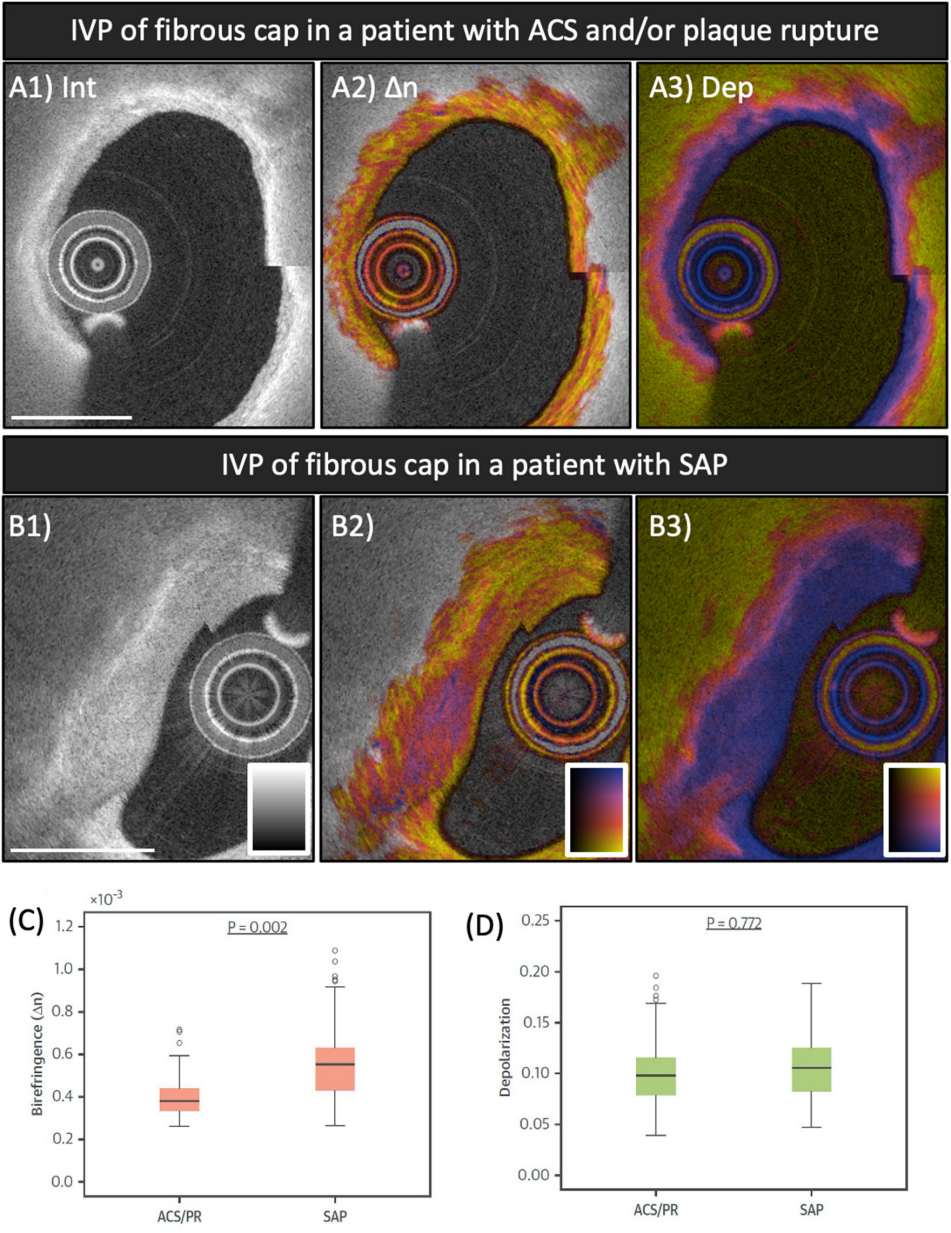
Polarization features of fibrous caps of culprit lesions in ACS and SAP patients. **(A1–A3)** IVP of the culprit lesion in a patient with ACS. **(B1–B3)** IVP of the target lesion in a patient with SAP. **(C,D)** Comparison of mean birefringence and depolarization measured in the segmented fibrous caps between patients with ACS/PR and SAP. Fibrous caps in ACS/PR patients featured significantly lower birefringence compared to those in SAP patients. ACS, acute coronary syndrome; IVP, intravascular polarimetry; PR, plaque rupture; SAP, stable angina pectoris. Reproduced from Otsuka et al. (35, 36).

Collagen is an extracellular matrix protein mainly synthesized by arterial SMC. Thick collagen fibers are known to impart mechanical integrity to fibrous caps (64). Collagen degradation by matrix-metalloproteinases plays a pivotal role in plaque destabilization (1). Because collagen is birefringent, evaluating birefringence can serve as unique metric for studying fibrous cap stability. In the first-in-human study of IVP, we further compared the polarization signatures measured locally in the fibrous caps of culprit lesions between patients with ACS and/or plaque rupture (PR) and patients with SAP (35). Figure 4 shows a representative IVP image of both categories and the comparison of the polarization properties measured across all fibrous caps in patients with ACS/PR and SAP. The fibrous caps in ACS/PR patients featured significantly lower birefringence than those in SAP patients (35). Based on previous observations from a microscopic PS-OCT study on human aortic plaques *ex vivo* (39), our observation is consistent with a lack of thick collagen fibers or layered arrays of SMCs in ACS/PR fibrous caps. Further research is warranted to investigate how fibrous cap birefringence influences the biomechanical factors that are associated with plaque rupture and subsequent thrombosis (65, 66). Furthermore, inflammatory activity mainly caused by macrophages contributes to the thinning of the fibrous cap covering a necrotic core and to precipitating plaque rupture (21, 67, 68). In a focal analysis of the thinnest part of the fibrous caps, we found that depolarization correlated positively with normalized standard deviation, a metric based on the reflection intensity signal that has been shown to indicate macrophage accumulation (69–71). Depolarization may help to improve identification of local macrophage accumulation within the fibrous caps and also to automatically delimit the cap border. This may provide robust assessment of fibrous cap thickness without discordance between observers. Despite the promise of the observations with IVP, it should be noted that the influence of microcalcifications on the polarization features as well as their appearance in conventional OCT remain unknown (54, 59). We speculate that the presence of microcalcifications causes a reduction of birefringence in fibrous caps, similar to a lack of well-organized collagen or SMCs. Combination of Micro-OCT imaging (72, 73) with polarimetry may help elucidate the influence of microcalcifications on the polarimetric signatures of coronary plaques.

Although plaque erosion remains challenging to diagnose *in vivo* with OCT, its apparent prevalence in autopsy studies has motivated clinical research focused on studying its pathobiology and on improving therapeutic strategies for patients with ACS caused by this second most common mechanism of coronary thrombosis (68, 74). According to histopathological findings, eroded plaques feature negatively remodeled lesions with SMCs embedded in a proteoglycan-rich extracellular matrix consisting of collagen type III, hyaluronan, and versican (17). While the presence of fibrillar collagen type III may contribute to birefringence, in the IVP pilot study, we observed that fibrous caps of patients with plaque rupture and non-rupture ACS exhibited lower birefringence than those of patients with SAP. Further research is needed to better understand whether IVP may be able to provide additional insights into the mechanisms of ACS caused by plaque erosion.

## Polarization Signatures of Acute and Organizing Thrombus

**Figure 5:**
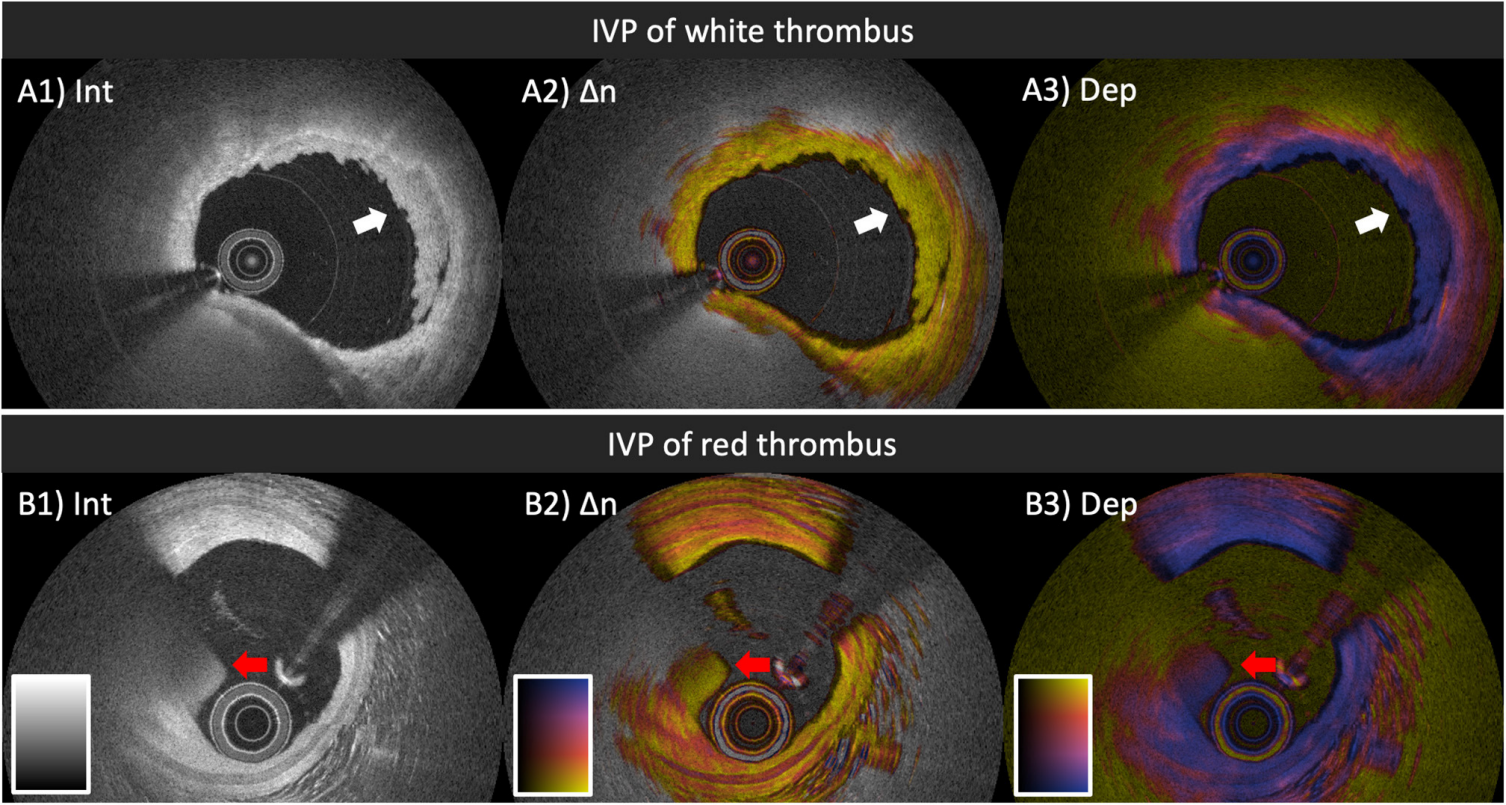
IVP of thrombi in patients with ACS. **(A1–A3)** White thrombus (white arrow) attached to the luminal surface, exhibiting homogeneous backscattering and low attenuation **(A1)**, paired with low birefringence **(A2)** and low depolarization **(A3)**. **(B1–B3)** Red thrombus (red arrow) protruding into the lumen, featuring high backscattering, and attenuation **(B1)**, together with relatively low birefringence **(B2)** and gradually increasing depolarization **(B3)**. IVP, intravascular polarimetry.

Conventional OCT enables the discrimination between white and red acute thrombus based on the attenuation of the intensity signal (Figures 5A1,B1). White, platelet-rich thrombus causes little backscattering and appears homogeneous with low signal attenuation (Figure 5A1), while red thrombus, mainly composed of erythrocytes, is strongly backscattering with a high signal attenuation (Figure 5B1) (54). In the study population of the first-in-human pilot study of IVP, we observed that white thrombus displayed low birefringence and depolarization (Figures 5A2,A3) (35), but did not encounter any red thrombus. In the second pilot study of IVP at the Erasmus University Medical Center (POLARIS-I registry), we observed a few red thrombi in the culprit lesions of ACS patients. In the limited number of examples, red thrombus exhibited low birefringence. The apparent increase in depolarization along depth is likely an artifact caused by the rapid decline of the intensity signal along depth within red thrombus (Figure 5B3). In the same patient pool, we imaged a suspected culprit vessel of a 69-year-old female patient presenting with non-ST-segment elevation myocardial infarction several days after onset of intermittent chest pain (45). The OCT intensity image revealed honeycomb-like structures in the culprit lesion, which is a well-known OCT-specific feature of recanalization in organized thrombus or chronic total occlusion (26). IVP revealed high birefringence in the honeycomb-like structures (45), suggesting the presence of collagen and SMCs, which should be expected for organizing and healing thrombus.

Extensive research has focused on the mechanisms underlying rapid progression of coronary artery stenosis (22). This is important for the identification of patients at higher risk of coronary ischemic or thrombotic events. It is now widely accepted that only a minority of plaque ruptures and erosions progress to ACS with clinical manifestation. Subsequent thrombus formation and organization, in which the intimal microvasculature plays a pivotal role, are key to understanding the mechanisms of successful or failed vascular healing (24). In a recent *ex vivo* study investigating the accuracy of OCT for diagnosing healed ruptured plaques, Shimokado et al. proposed the presence of layered structures in the conventional OCT intensity signal as a predictor of histologically determined healed ruptures of coronary plaques (75). Furthermore, clinical studies have demonstrated that layered structure in superficial plaques identified by OCT are associated with plaque vulnerability or rapid progression of coronary artery stenosis (10, 76), although there remains some controversy and another study found similar features to indicate enhanced plaque stability (23). Despite the importance of assessing the healing process of coronary thrombus, the conventional OCT intensity image provides limited insight into the process of thrombus organization. The polarization signatures measured with IVP have the potential to characterize the thrombus organization and healing process, since the deposition of collagen type III and later replacement with collagen type I should result in a change in birefringence (23, 77, 78). Assessment of coronary atherosclerosis with IVP *in vivo* may help investigate the mechanisms of impaired vascular healing that lead to ACS.

## Polarization Signatures of Tissue Response After Stent Implantation

**Figure 6:**
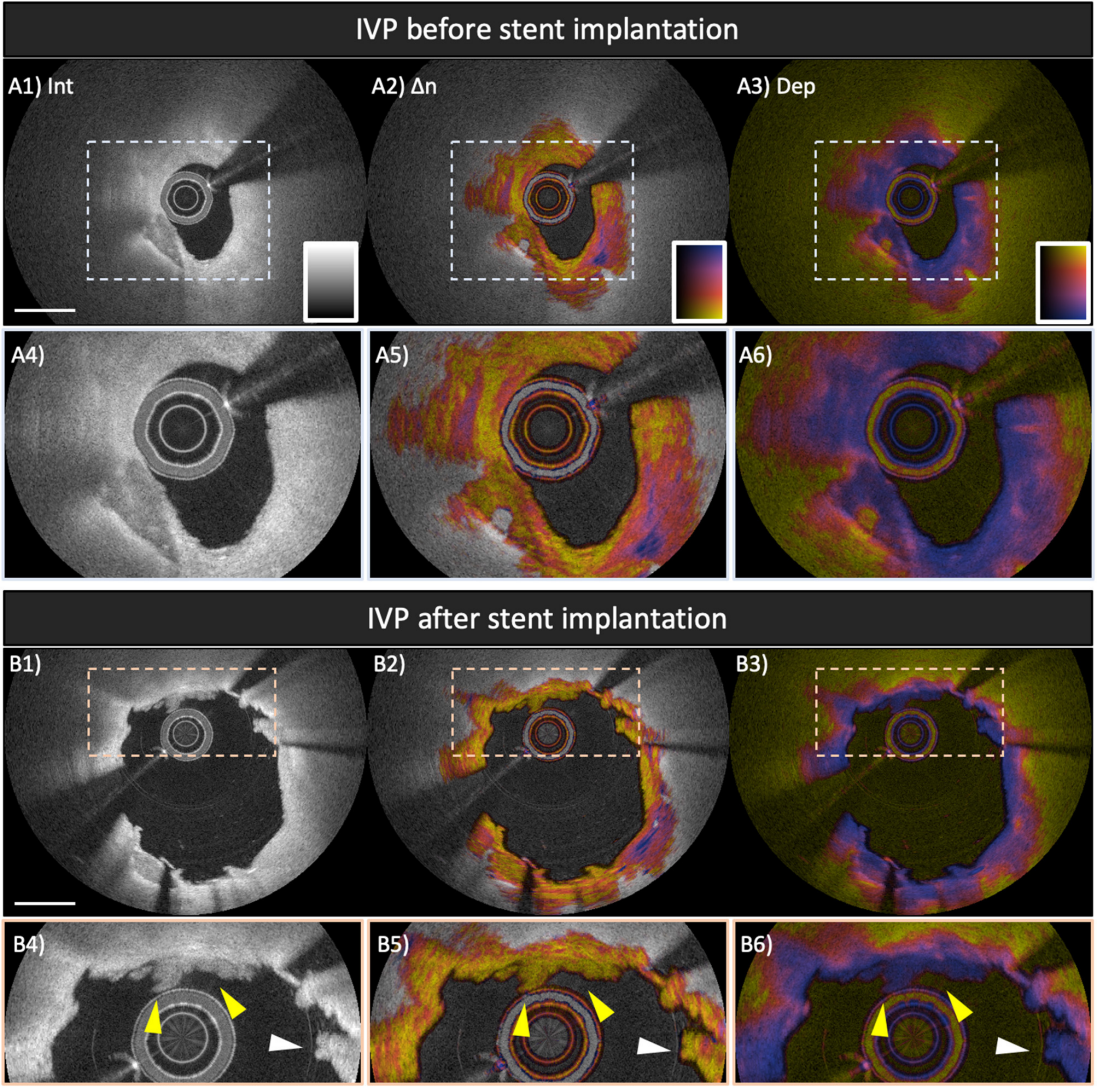
Polarization features pre- and post-stent implantation in the culprit lesion of a patient with non-ST-elevation myocardial infarction. Broken lines in **(A1–A3)** and **(B1–B3)** indicate magnified views shown in **(A4–A6)** and **(B4–B6)**, respectively. **(A1,A4)** IVP of the culprit lesion showing a lipid-rich plaque with calcification at 7 o'clock **(A2, A5)** Birefringence image reveals highly birefringent tissues from 3 to 6 o'clock within the fibrous cap, suggesting well-organized fibrous tissues. **(B1–B6)** Post-stent IVP of the culprit lesion. **(B1,B4)** The reflection intensity images visualize tissue protrusion or prolapse (white arrowhead) and in-stent thrombus formation (yellow arrowheads). IVP clearly reveals in-stent thrombus showing low birefringence and low depolarization, which is the typical IVP appearance of white thrombus **(B2,B5)**. Scale bars indicate 1 mm. IVP, intravascular polarimetry.

The high-resolution of OCT provides detailed visualization of the tissue response to stent implantation, together with the assessment of stent apposition and expansion (79, 80). In addition to the plaque morphology before stent implantation, post-stent OCT features including stent-edge dissection, in-stent thrombus, and protrusion have been shown to be associated with peri-procedural myocardial injury/infarction (58, 81–83) and long-term device-oriented coronary events (84). Figure 6 shows pre- and post-stent IVP images of the culprit lesion in a non-ST-segment elevation myocardial infarction patient. The intensity image visualizes tissue protrusion or prolapse (white arrowhead in Figure 6B4) and in-stent thrombus formation (yellow arrow heads in magnified view of Figure 6B4). The IVP image complements the intensity appearance of the in-stent thrombus with low birefringence and depolarization, which is, to the best of our knowledge, the typical IVP appearance of white thrombus. In comparison, the prolapsed tissue contains an area of increased birefringence. Given the additional tissue contrast provided by IVP, it may help to reduce the discordance in image interpretation between observers and may also offer additional insight into the vascular injury and the ensuing healing process of the coronary arterial wall related to stent implantation.

**Figure 7:**
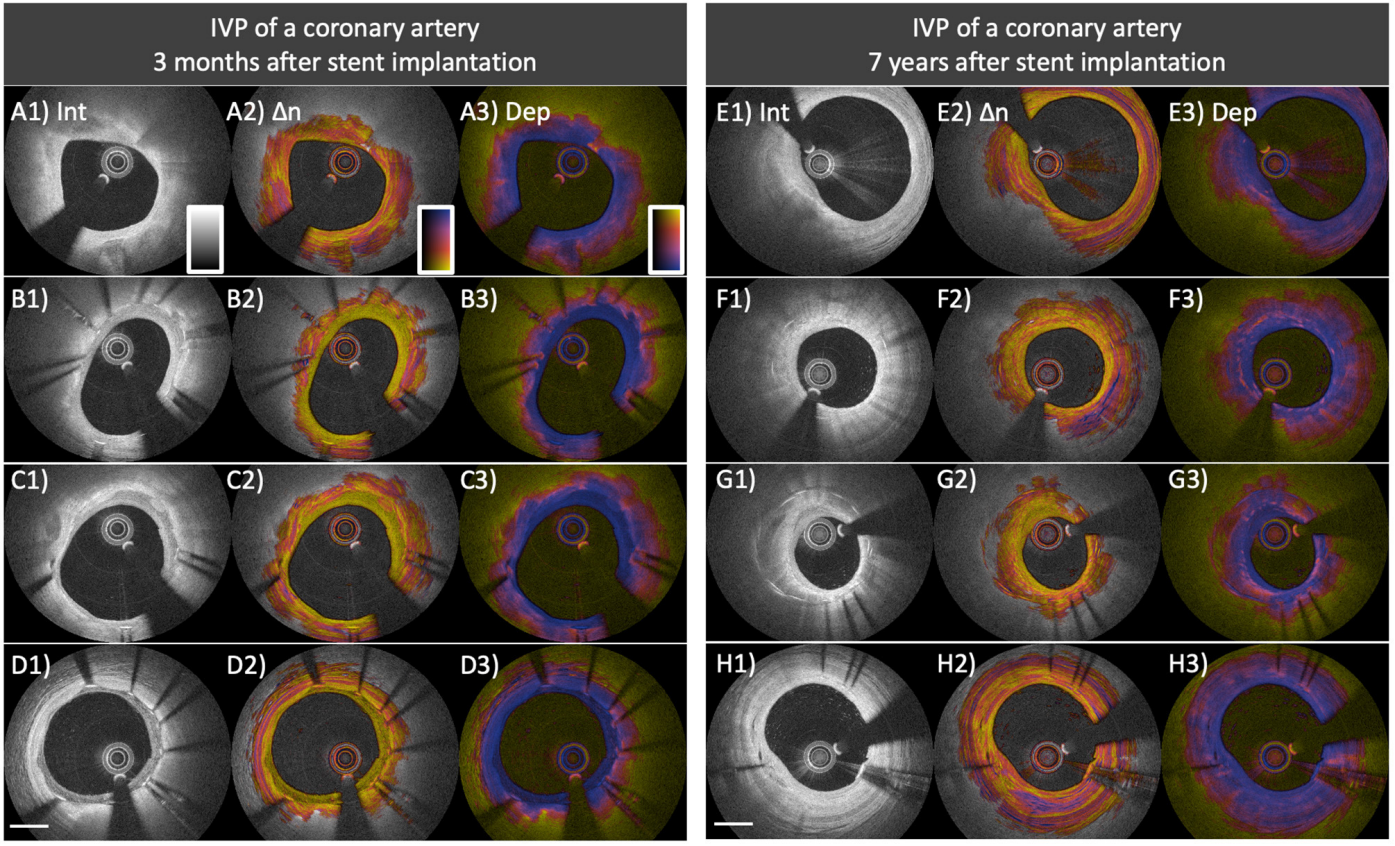
Distinct polarization features of neointima and neoatherosclerosis. **(A–D)** IVP of a coronary artery 3 months after everolimus-eluting stent implantation. **(A1)** Native fibro-calcified plaque proximal of the stented segment. **(B1–D1)** The intensity images within the stented area show early neointima with high **(B1)** to low **(D1)** intensity signal. **(B2–D2)** Birefringence images reveal that early neointima exhibits low birefringence compared to that of the underlying native lesion. **(B3–D3)** Depolarization in early neointimal tissues remains low. **(E–H)** IVP of a coronary artery 7 years after paclitaxel-eluting stent implantation. **(F1)** Native fibro-calcified plaque proximal of the stented segment. **(F1,G1)** The intensity images show neoatherosclerosis featuring calcification, macrophage accumulation, and layered structure. **(F2,G2)** Birefringence images reveal lowly birefringent layered structures close to the surface of the arterial lumen. The layered structure in **(G2)** exhibits lower birefringence compared to the underlying neoatherosclerotic region. **(F3,G3)** Macrophage accumulations within neoatherosclerosis cause pronounced depolarization. **(H2)** The birefringence image of the late neointima without neoatherosclerosis reveals relatively high birefringence, comparable to that of underlying native plaque and the native plaque proximal of the stented segment **(E2)**.

Long-term vascular tissue response to stent implantation is of importance for assessing the risk of stent failure, such as in-stent restenosis and stent thrombosis (25, 85, 86). In a swine study investigating vascular response to bioresorbable vascular scaffold implantation, polarization properties measured with PS-OCT reflected tissue organization and inflammation within the neointima (87). Figure 7 shows several examples of IVP cross-sections in previously stented coronary arteries encountered in the patient cohorts of our pilot studies. Compared to native intima (Figure 7A2) it could be expected that the early neointima in drug-eluting stents exhibits lower birefringence (B2, C2, and D2 in Figure 7), owing to a drug-induced suppression of collagen content and SMCs (88). In contrast, the late neointima (7 years after stent implantation, Figure 7H2) shows relatively higher birefringence which is comparable to that of native plaques (Figure 7E2). A recent study conducted by Suna et al. demonstrated that aggrecan, a major extracellular matrix component of cartilaginous tissues that confers resistance to compression, plays a critical role in arterial remodeling after the implantation of drug-eluting stents (DES) (89). Furthermore, the same study showed that the extracellular matrix of the neointima within DES contained less collagen type I, III, and V than the neointima in bare metal stents (89). These findings offer a plausible explanation for the IVP appearance of early neointimal tissues in Figure 7, featuring very low birefringence. In addition, neoatherosclerosis (Figures 7F2,G2) features a layered pattern with relatively low birefringence. Histopathological studies have demonstrated that ECM deposition and migration of SMC contribute to the healing of thrombus and may result in rapid progression of luminal narrowing leading to ISR (25). Similar to native coronary atherosclerosis, assessing tissue composition with IVP may provide insight into the failure of vascular healing also after stent implantation.

While birefringence serves as a marker of the presence of collagen content and SMCs, depolarization corresponds to the presence of macrophage accumulation and the presence of a lipid/necrotic core (34, 36). Neoatherosclerosis is characterized by the presence of lipid-laden macrophages (85) and depolarization may offer a convenient quantitative metric for its identification (Figures 7F2,G2). We anticipate that IVP will advance our understanding of neoatherosclerosis and stent thrombosis, which in turn could improve the risk assessment and patient prognosis in a more personalized and precise manner.

## Future Perspectives

Additional histopathological studies will be needed to clarify in more detail the tissue and plaque morphology that underlies the observed polarization features and improve the interpretation of the quantitative polarization metrics. For instance, investigation of the mechanisms causing depolarization in lipid-rich and necrotic core material may offer a differentiating feature to enable accurate identification of fibroatheromas. Toward a similar goal, the combined use of IVUS and OCT has been shown to offer more accurate diagnosis of TCFAs than each modality alone (52, 90). A single catheter that integrates IVUS and OCT provides intrinsically co-registered cross-sectional images from both modalities (91–93). Combination of IVUS and IVP would be feasible using the same dual-modality catheters and offer the advantages of visualizing the entire lesion-depth with IVUS and more superficial fine structural details with OCT, together with the improved tissue characterization of IVP. Another strategy toward increased compositional sensitivity could be to combine polarization analysis with detecting spectral absorption features that have been shown to help in the detection of lipid (94–96).

**Figure 8:**
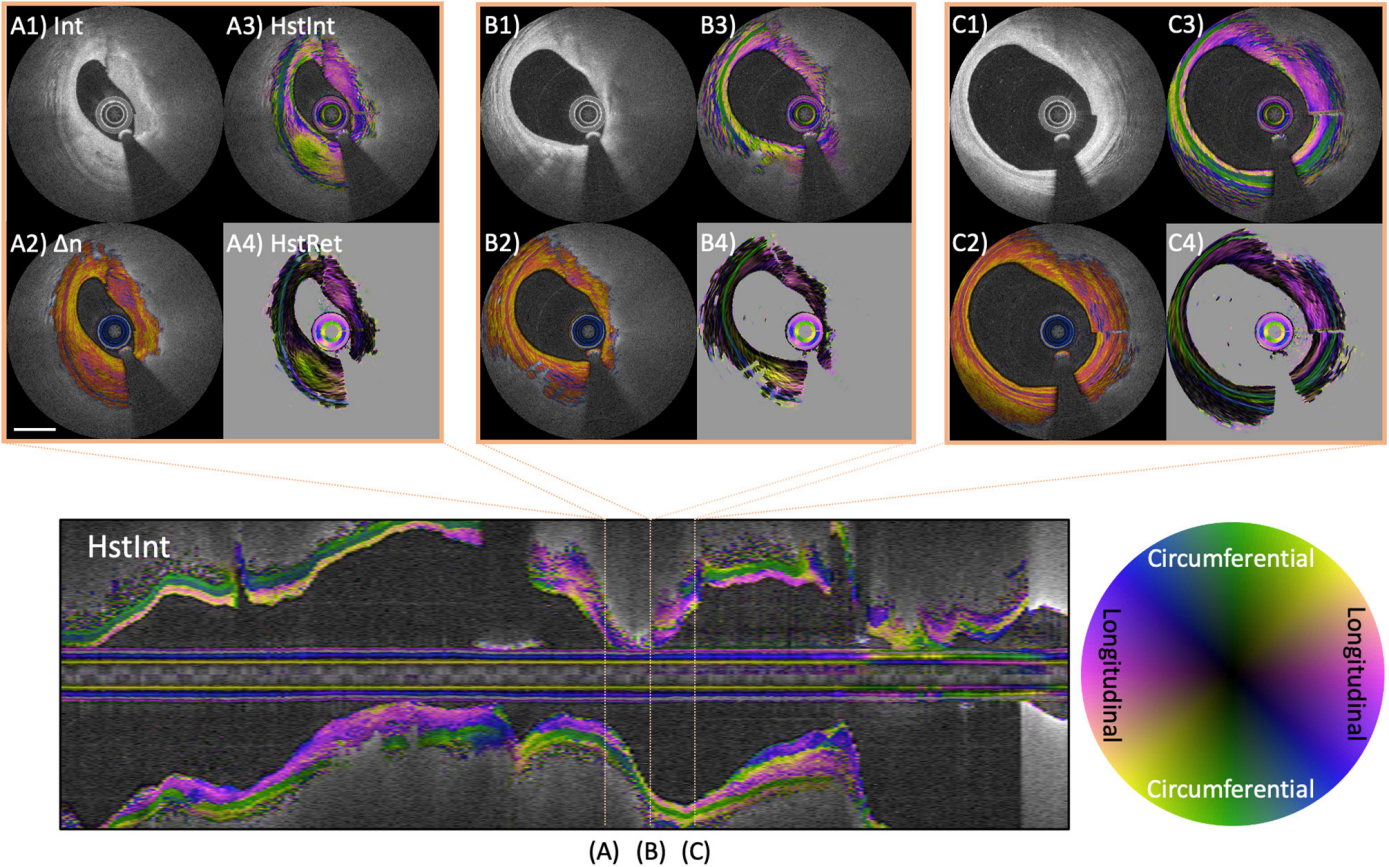
Optic axis orientation in coronary atherosclerotic lesion. Longitudinal image displays the optic axis orientation overlaid on intensity of a coronary artery in a patient with stable angina pectoris. Color indicates axis orientation and brightness specifies intensity. **(A–C)** Three individual cross-sections, showing conventional intensity **(A1,B1,C1)**, birefringence **(A2,B2,C2)**, and depth-resolved optic axis overlaid on intensity **(A3,B3,C3)** or birefringence **(A4,B4,C4)**. The tunica media features green color, indicating circumferential orientation. **(A3,A4)** Heterogeneous optic axis orientation of the intimal fibrous tissue. The fibrous cap in **(B3,B4)** and fibrous areas in **(C3,C4)** feature longitudinal orientation of the optic axis. Scale bar indicates 1 mm.

In a further development, we extended our reconstruction method to obtain not only the scalar amount of birefringence, but also its optic axis orientation as a function of depth in the vessel wall (97). The optic axis indicates the physical orientation of the fibrillar tissue structures giving rise to birefringence. More specifically, the optic axis orientation indicates the azimuthal direction of the fibrillar components, i.e., the orientation of their projection into a plane orthogonal to the beam axis. While the measured birefringence depends on the alignment of the fibrillar components with the beam direction, the full three-dimensional orientation of the fiber orientation cannot currently be recovered. Conveniently, in the arterial wall of coronary arteries the fibrillar tissue components can be assumed to be naturally oriented quite orthogonal to the OCT probing beam. As shown in Figure 8, the optic axis of fibrous tissues within the intima corresponding to adaptive intimal thickening aligns longitudinally along the vessel direction, while the tunica media features circumferential orientation. In advanced atherosclerotic lesions, the optic axis orientation frequently revealed distinct tissue layers that appear continuous in conventional OCT images and that also feature remarkably uniform scalar birefringence (Figure 8). Optic axis orientation may provide unique and mechanistic insight into the progression of coronary atherosclerosis and the tissue response to stent implantation. The vascular healing response is thought to lead to a distinct orientation of the organizing thrombus and the deposited collagen, producing noticeable features in the optic axis orientation. Improving the robustness of the reconstruction of optic axis orientation and histopathologic validation of this metric are still needed.

The *ex vivo* and clinical IVP studies thus far have established that IVP provides robust measurements of polarization properties of coronary atherosclerosis. The polarization signatures enable more detailed tissue characterization than conventional OCT. An important benefit is the quantitative nature of the polarization metrics, which will facilitate their standardization and assist OCT image interpretation. Despite our efforts of developing an intuitive display, the multiple signal channels of IVP are challenging to visualize and interpret. However, they offer the promising opportunity to leverage the powerful capability of state-of-the-art deep learning routines. Advanced machine learning techniques are poised to radically impact imaging-based methods. They can provide image segmentation and interpretation that is too time consuming to be performed manually in real-time but would furnish critical feedback to guide interventions. Artificial neural networks have already been adapted for robust lumen segmentation (98), for classification of intracoronary OCT images (99), and for improved stent strut detection (100). We anticipate that the additional contrast available to IVP paired with advanced deep learning routines will significantly improve the level of detail that can be automatically identified and extracted from IVP pullbacks and will enable robust automated segmentation and classification of coronary atherosclerotic lesions.

Lastly, it is worth pointing out that the spatial resolution of OCT by far exceeds the dimension of individual collagen fibrils and even fibers, or actin and myosin filaments. At best, OCT resolution may be sufficient to resolve collagen bundles. IVP provides insight into the organization and arrangement of tissue fibrils and filaments on a sub-resolution scale by detecting the resulting tissue birefringence. The thickness, density, and linearity of collagen fibrils in their hierarchical organization within the fibrous tissue of the intima all contribute to the observed birefringence. Furthermore, birefringence is non-specific and can also arise from the combined effect of different fibrillar tissue components. In a similar way, multiple mechanisms contribute to tissue depolarization. Current efforts are aimed at elucidating specific polarization effects in the attempt to disentangle the various contributions. Microscopic spatial resolution would help to identify specific tissue substrates but remains incompatible with imaging in a clinical setting.

## Conclusions

IVP is an extension of conventional OCT that measures polarization properties of the coronary arterial wall through standard commercial imaging catheters and without altering the imaging procedure. Birefringence relates to the collagen and smooth muscle content, which is an important determinant of plaque stability and vascular healing. Depolarization highlights the presence of lipid and macrophages. The quantitative nature of the polarization metrics offers a pathway beyond the qualitative interpretation of conventional tomograms and toward automated identification of critical features, such as fibrous cap thickness. The improved insight into plaque composition afforded by IVP provides new opportunities to investigate disease progression and development of ACS. IVP may provide surrogate markers for improving risk stratification of patients with coronary artery disease.

## Author Contributions

All authors have participated in the drafting of the manuscript and have approved the final version of the manuscript.

## Conflict of Interest

Massachusetts General Hospital and the Erasmus University Medical Center have patent licensing arrangements with Terumo Corporation. BB and MV have the right to receive royalties as part of the licensing arrangements. The remaining authors declare that the research was conducted in the absence of any commercial or financial relationships that could be construed as a potential conflict of interest.

## Footnotes

^1^^*^ Throughout this manuscript we generalize OFDI – a specific second-generation implementation of optical coherence tomography – to OCT, the general imaging method that includes all implementations of OCT, whenever suitable.
